# G3F: Global, Multidimensional Spectral Regression Analysis

**DOI:** 10.21105/joss.01629

**Published:** 2019-08-27

**Authors:** Allison M. Stettler, Christopher W. John, Yegor D. Proshlyakov, Denis A. Proshlyakov

**Affiliations:** 1 Department of Chemistry, Michigan State University

## Summary

### Rationale:

Multi-dimensional non-linear global regression permits the investigation of quantitative relationships in complex datasets and to examine validity of proposed models. However, traditional multi-dimensional regression requires predictable variation of all parameters along every fitted dimension. This constraint may be difficult to satisfy, for example:

- When noise in any particular dimension exceeds the signal of interest, such as with large variation of baselines due to thermal fluctuations, sample variability, or other interference.
- When the common signal is too complex for rational description using a reduced set of variables, such as encountered with multiple bands in high-resolution spectra. The unknown spectra of one or more species, involved in a predictable process, may be better described by a large set of independent coefficients that vary between discrete sampled energies (frequency, wavelength, mass, charge, etc.) than by a limited set of Gaussian or Lorentzian peaks.
- When observables cannot be accurately described using a trivial band shape or a distribution such as, for example, when a normally distributed signal is broadened by spectral resolution with a rectangular profile.
- When spectral overlap does not allow achieving experimental resolution of several distinct signals, especially when their individual properties are not known.
- When describing intensity in an inhomogeneous kinetic process, a process with an unknown kinetics, or when the kinetics may be too complex to be described by a reasonable number of phases.

Common to these examples is the need to describe a multi-dimensional experimental dataset not only using globally invariable parameters (frequencies, temperature, rate constants), but with variable vectors of parameters that are applicable to one or more dimensions (local parameters).

### Concept:

The G3F package for IgorPro was initially developed to simultaneously analyze vibrational spectra of multiple isotopomers with overlapping modes in time-resolved Raman studies on enzyme TauD, where simple improvement of signal via time averaging was not possible (Grzyska, Appelman, Hausinger, & Proshlyakov, 2010). Analysis of a frequency vs. time 2D dataset with uniform properties of predictable vibrational bandshapes (global) and unknown speciation plots (local to each time point) allowed resolution of superimposed vibrations of two different species while improving resolution via signal sharing between spectra. Also included were polynomial baselines, which varied between spectra. A similar approach was later used in the analysis of Raman spectra of methane monooxygenase (Banerjee, Proshlyakov, Lipscomb, & Proshlyakov, 2015). In more recent studies, it was used to obtain completely unknown difference spectra of a redox transition with an unknown potential over a variable polynomial background (John & Proshlyakov, 2019; John, Swain, Hausinger, & Proshlyakov, 2019). In this case, analysis involved two orthogonal parametric vectors (frequency-dependent amplitudes vs. potential-dependent populations).

### Implementation:

G3F uses IgorPro’s internal non-linear regression engine to recursively minimize residual error. G3F handles folding of complex global and local data into a form suitable for the built-in engine. Fitted data can be a 2D (columns) or 3D (layers) matrix with each dimension described by its own independent variable (calibration) and an optional set of zero or more fitted parametric vectors (row, column, and layer local variables). In addition to the layer-uniform row and column variables, G3F allows using layer-specific local fitted variables, LayRow and LayColumn. In line with the conventional fitting, G3F implements concepts of subranges and data masks in each of three fitted dimensions. To reduce computational load of covariance analysis over large sets of local variables (thousands), G3F introduces the concept of data thinning, which allows uesrs to box-average or drop equally-spaced data points along different dimensions, independently of each other.

While most of the preparation, verification, and reporting are transparent to the end user, G3F requires minimal knowledge of the IgorPro programming language for the user to be able to program calculation of the desired model. G3F uses a GUI control panel that allows the user to select a conforming fitting function. G3F recognizes multiple templates of user-supplied fitting functions depending on the dimensionality of data and the need for additional parameters, as described in the API and User Manual.

To allow analysis of complex phenomena, such as continuous-time Markov processes (Anderson & Kurtz, 2011; C. Zhang et al., 2019), for example, G3F supports a bi-phasic approach where the process is calculated first, using only global parameters, and local observable parameters are fitted to this process second. Numerically integrating large sets of differential equations that describe multi-step chemical models or predict transient responses to irregular changes in the independent variable, for example, is typically more computationally demanding than calculating spectral signatures of one or more components. Such numerical integrations are independent of the subsequent frequency-dependent analysis of the observable and, therefore, do not need to be repeated for each sampled frequency. Once generated, the process description can be re-used over multiple iterations until any of global parameters that define the process change, resulting in a dramatic reduction of computational load. Process generation and local parameters fitting are also accomplished via user-supplied templated functions.

Internally, G3F uses the concept of proxy functions to implement user-supplied models of data. From an IgorPro standpoint, the same proxy function is always executed for every fitting iteration. This proxy function analyzes the supplied data configuration, identifies the appropriate template function, matches it to the specified user function, and transfers code execution to it, along with the set of current parameters, for data calculation. Covariance analysis and error minimization are performed internally by IgorPro. The use of proxy functions allows assembling most of the G3F code in an independent module, de-cluttering the general procedure namespace. Calculations in direct fitting and process (where possible) modes are parallelized.

In addition to handling data folding and transferring data to the fitting function, G3F includes a number of utilities helpful in the process of multidimensional fitting and the analysis of results. G3F allows overriding holds over subsets of dimensional variables or automatically alternate fitting of such subsets, which can greatly facilitate convergence from the initial guesses of local parameters that are far from optimal. G3F provides several options for obtaining visual feedback of fitness of model to the experimental data.

## Supplementary Material

Software repository
