# Supplementary figures and images for: G3F: Global, Multidimensional Spectral Regression Analysis
Source: J Open Source Softw. Author manuscript; Available in PMC 2020 Mar 24. (PMC7093062; doi:10.21105/joss.01629)

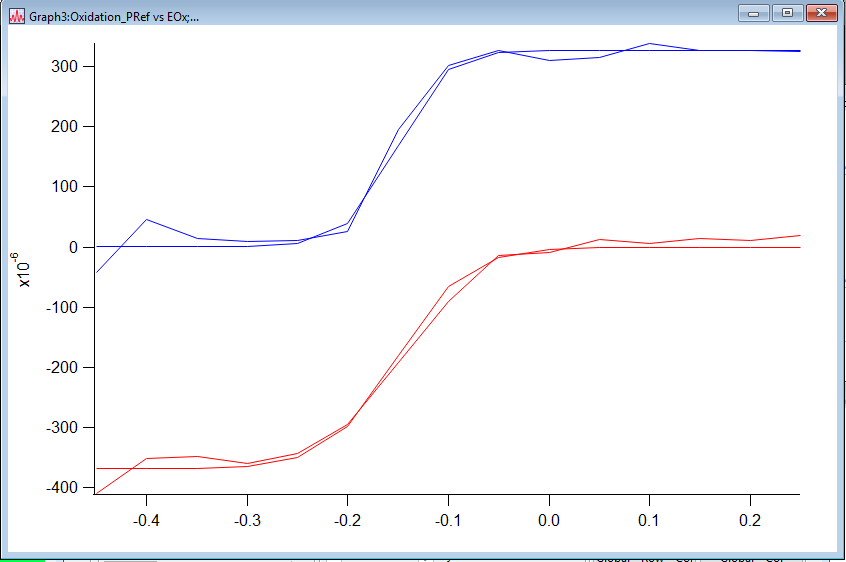

Supplement: Software repository [file NIHMS1559313-supplement-Software_repository.zip › G3F-master/Demo/DemoPictures/Pic_10_Demo.png]

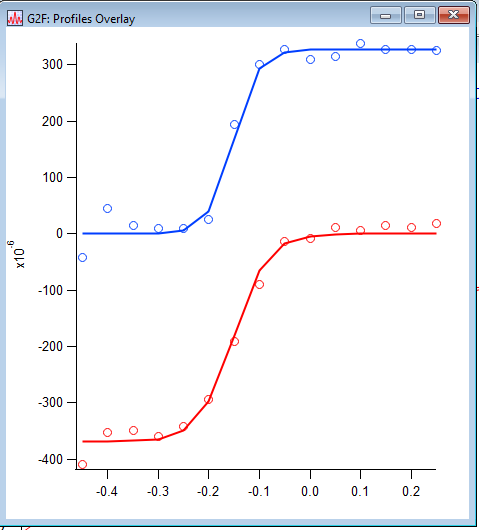

Supplement: Software repository [file NIHMS1559313-supplement-Software_repository.zip › G3F-master/Demo/DemoPictures/Pic_11_Demo.png]

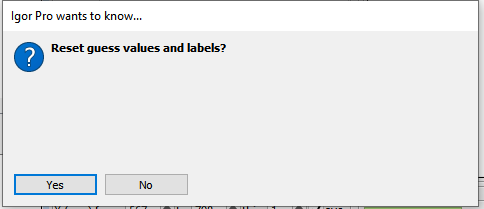

Supplement: Software repository [file NIHMS1559313-supplement-Software_repository.zip › G3F-master/Demo/DemoPictures/Pic_12_Demo.PNG]

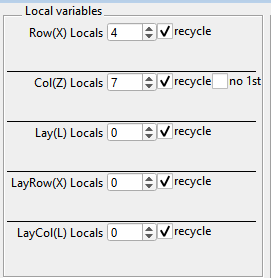

Supplement: Software repository [file NIHMS1559313-supplement-Software_repository.zip › G3F-master/Demo/DemoPictures/Pic_13_Demo.PNG]

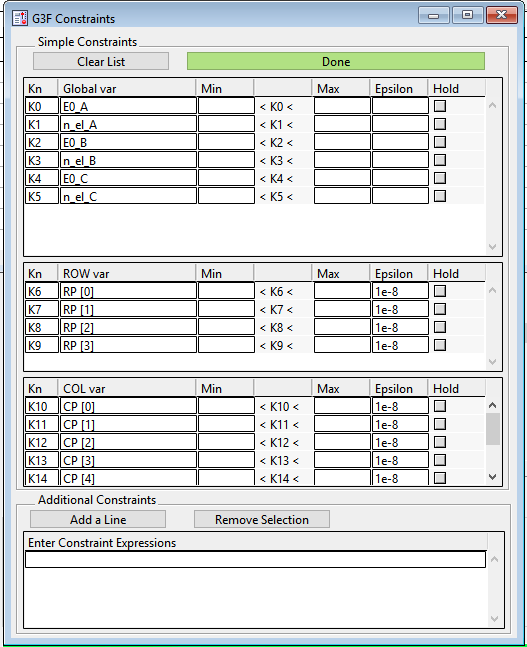

Supplement: Software repository [file NIHMS1559313-supplement-Software_repository.zip › G3F-master/Demo/DemoPictures/Pic_14_Demo.PNG]

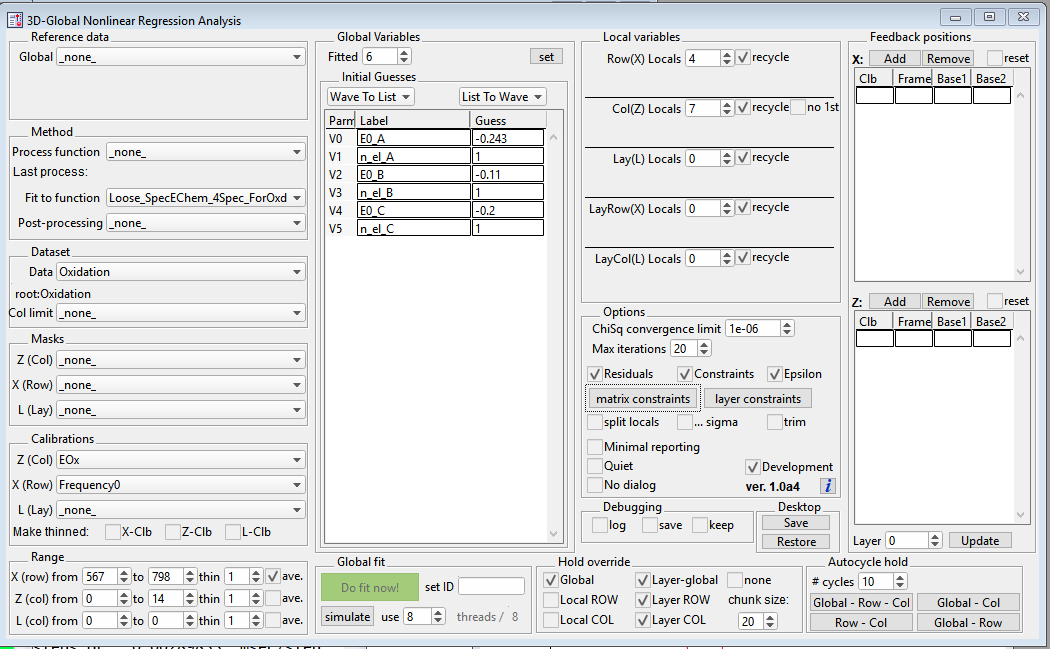

Supplement: Software repository [file NIHMS1559313-supplement-Software_repository.zip › G3F-master/Demo/DemoPictures/Pic_15_Demo.PNG]

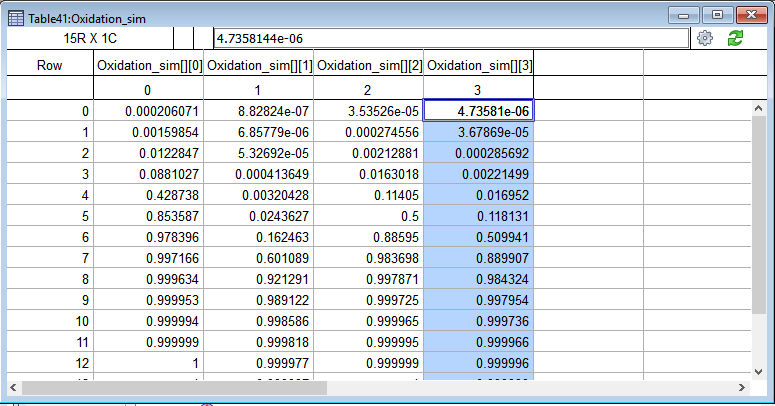

Supplement: Software repository [file NIHMS1559313-supplement-Software_repository.zip › G3F-master/Demo/DemoPictures/Pic_16_Demo.PNG]

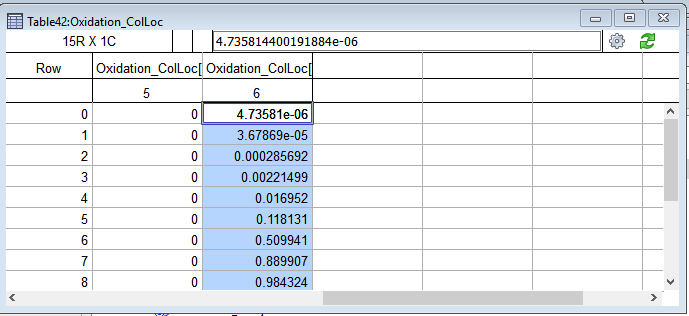

Supplement: Software repository [file NIHMS1559313-supplement-Software_repository.zip › G3F-master/Demo/DemoPictures/Pic_17_Demo.PNG]

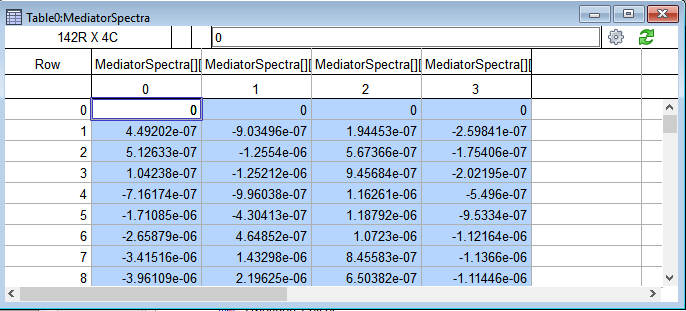

Supplement: Software repository [file NIHMS1559313-supplement-Software_repository.zip › G3F-master/Demo/DemoPictures/Pic_18_Demo.PNG]

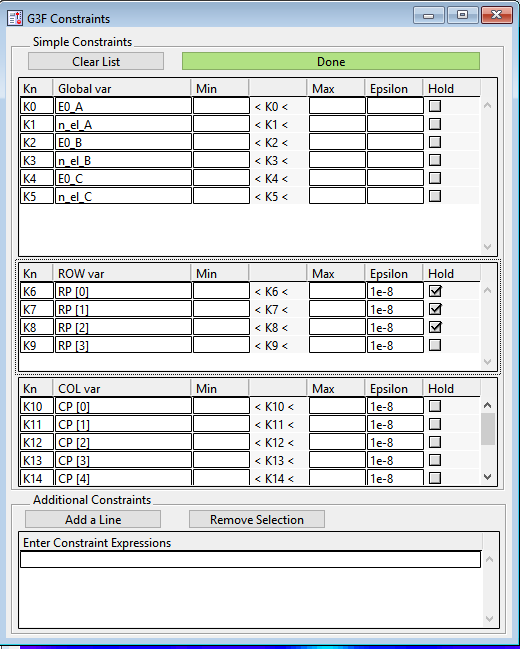

Supplement: Software repository [file NIHMS1559313-supplement-Software_repository.zip › G3F-master/Demo/DemoPictures/Pic_19_Demo.PNG]

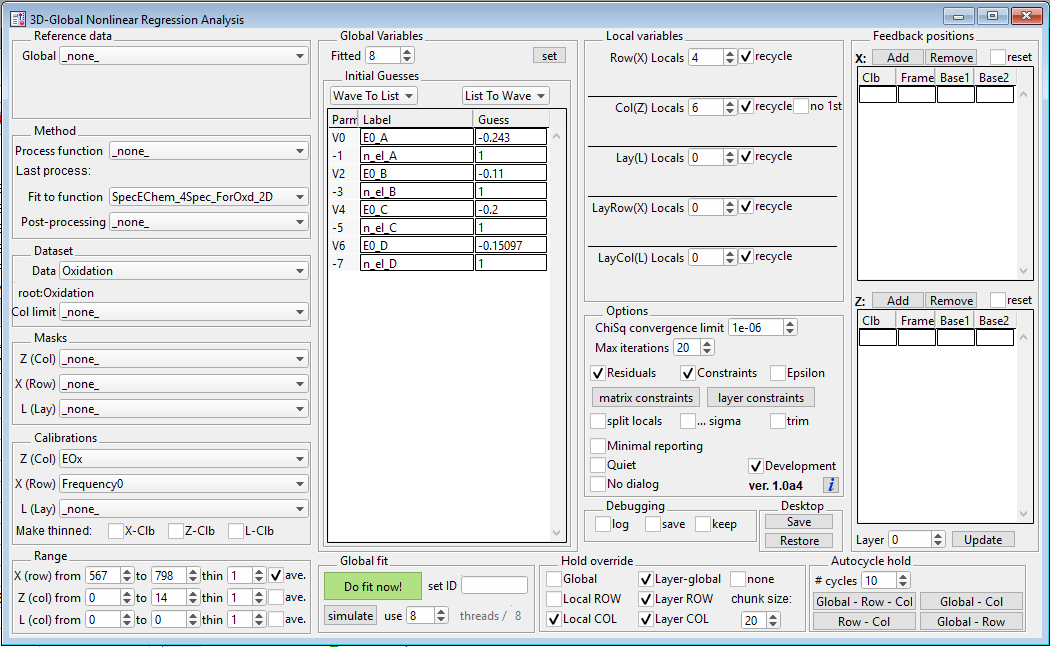

Supplement: Software repository [file NIHMS1559313-supplement-Software_repository.zip › G3F-master/Demo/DemoPictures/Pic_1_Demo.png]

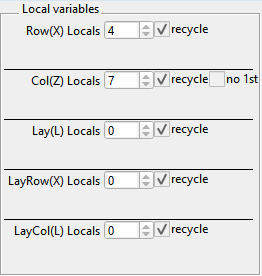

Supplement: Software repository [file NIHMS1559313-supplement-Software_repository.zip › G3F-master/Demo/DemoPictures/Pic_20_Demo.PNG]

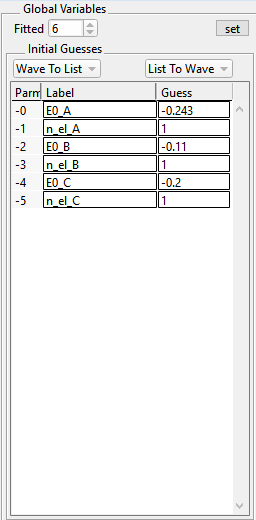

Supplement: Software repository [file NIHMS1559313-supplement-Software_repository.zip › G3F-master/Demo/DemoPictures/Pic_21_Demo.PNG]

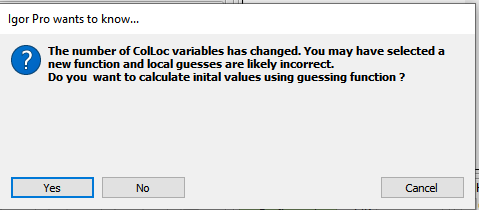

Supplement: Software repository [file NIHMS1559313-supplement-Software_repository.zip › G3F-master/Demo/DemoPictures/Pic_22_Demo.PNG]

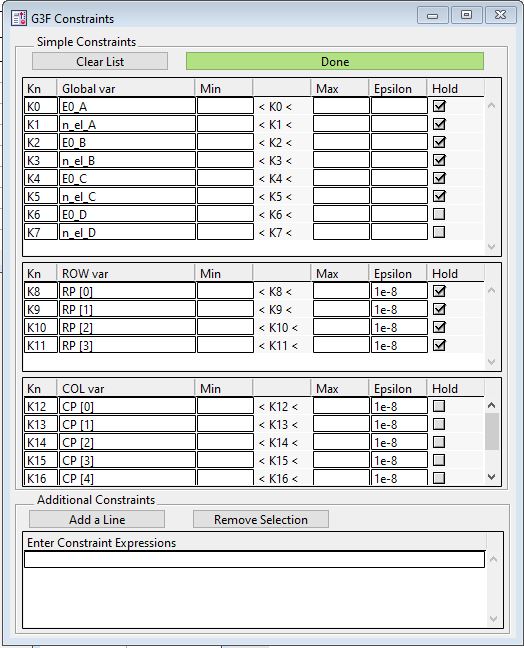

Supplement: Software repository [file NIHMS1559313-supplement-Software_repository.zip › G3F-master/Demo/DemoPictures/Pic_23_Demo.JPG]

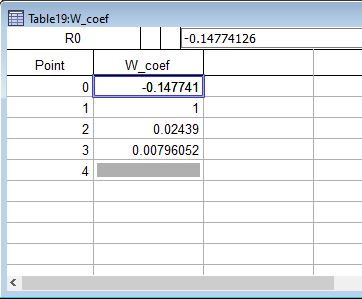

Supplement: Software repository [file NIHMS1559313-supplement-Software_repository.zip › G3F-master/Demo/DemoPictures/Pic_24_Demo.JPG]

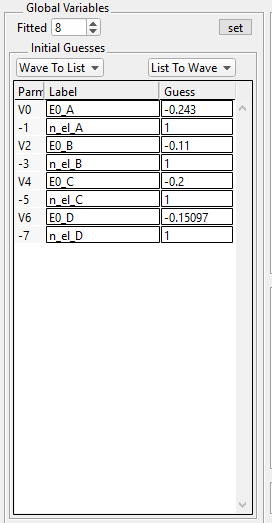

Supplement: Software repository [file NIHMS1559313-supplement-Software_repository.zip › G3F-master/Demo/DemoPictures/Pic_2_Demo.png]

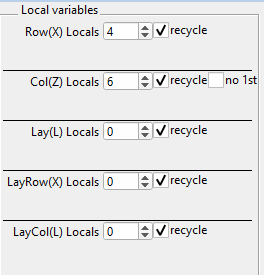

Supplement: Software repository [file NIHMS1559313-supplement-Software_repository.zip › G3F-master/Demo/DemoPictures/Pic_3_Demo.png]

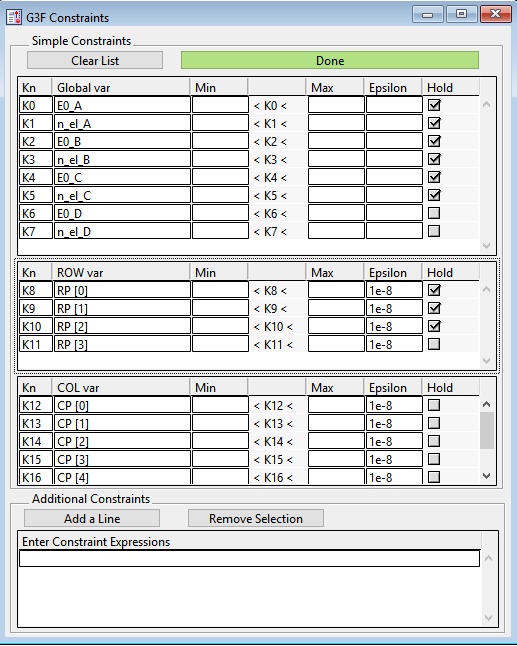

Supplement: Software repository [file NIHMS1559313-supplement-Software_repository.zip › G3F-master/Demo/DemoPictures/Pic_4_Demo.png]

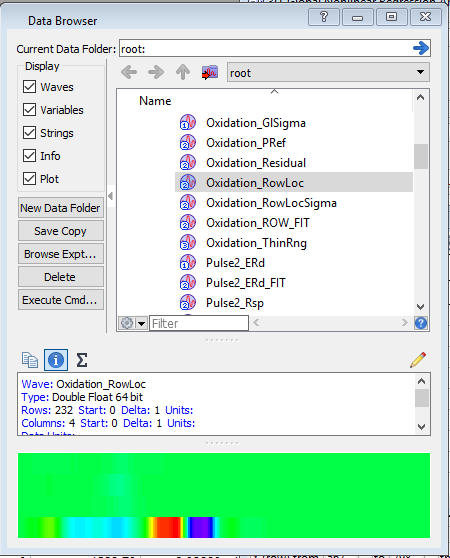

Supplement: Software repository [file NIHMS1559313-supplement-Software_repository.zip › G3F-master/Demo/DemoPictures/Pic_5_Demo.png]

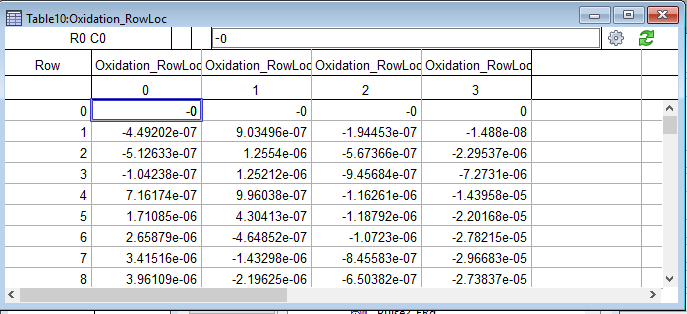

Supplement: Software repository [file NIHMS1559313-supplement-Software_repository.zip › G3F-master/Demo/DemoPictures/Pic_6_Demo.png]

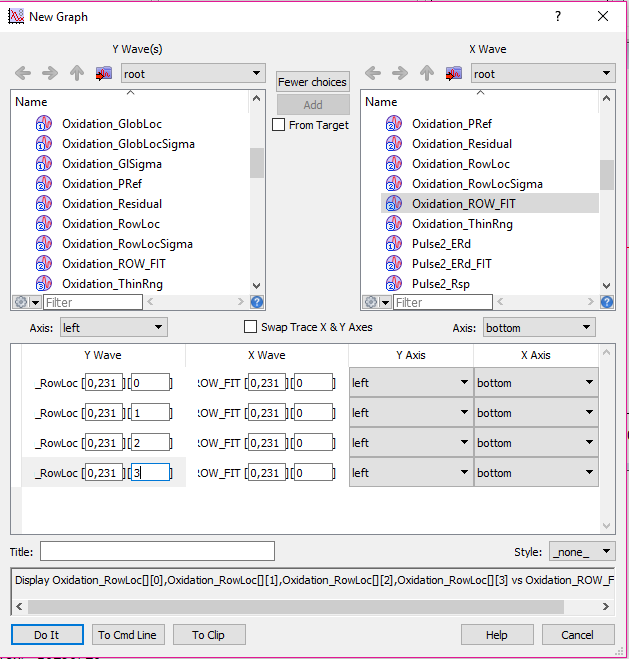

Supplement: Software repository [file NIHMS1559313-supplement-Software_repository.zip › G3F-master/Demo/DemoPictures/Pic_7_Demo.png]

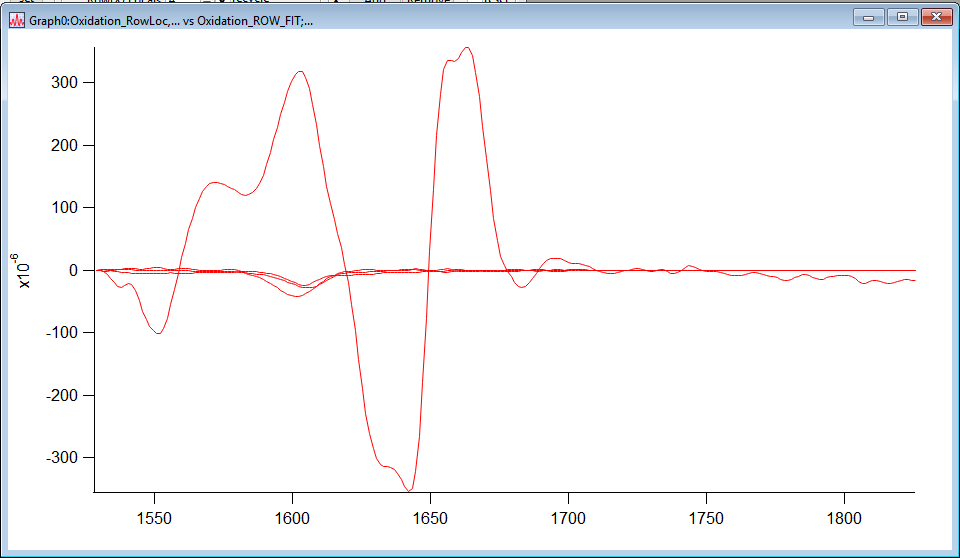

Supplement: Software repository [file NIHMS1559313-supplement-Software_repository.zip › G3F-master/Demo/DemoPictures/Pic_8_Demo.png]

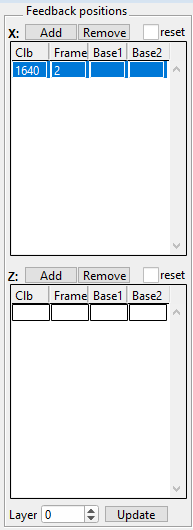

Supplement: Software repository [file NIHMS1559313-supplement-Software_repository.zip › G3F-master/Demo/DemoPictures/Pic_9_Demo.png]

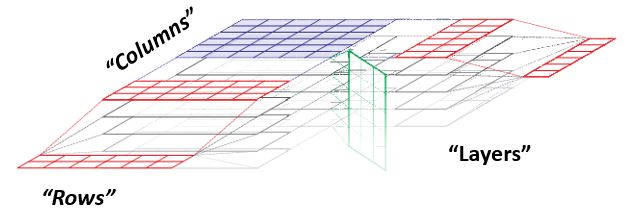

Supplement: Software repository [file NIHMS1559313-supplement-Software_repository.zip › G3F-master/Docs/G3FManualPictures/3D_DataStructure_A.JPG]

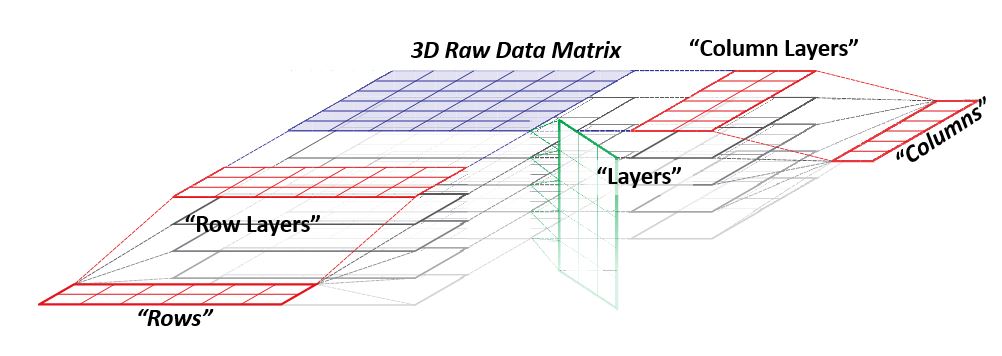

Supplement: Software repository [file NIHMS1559313-supplement-Software_repository.zip › G3F-master/Docs/G3FManualPictures/3D_DataStructure_B.JPG]

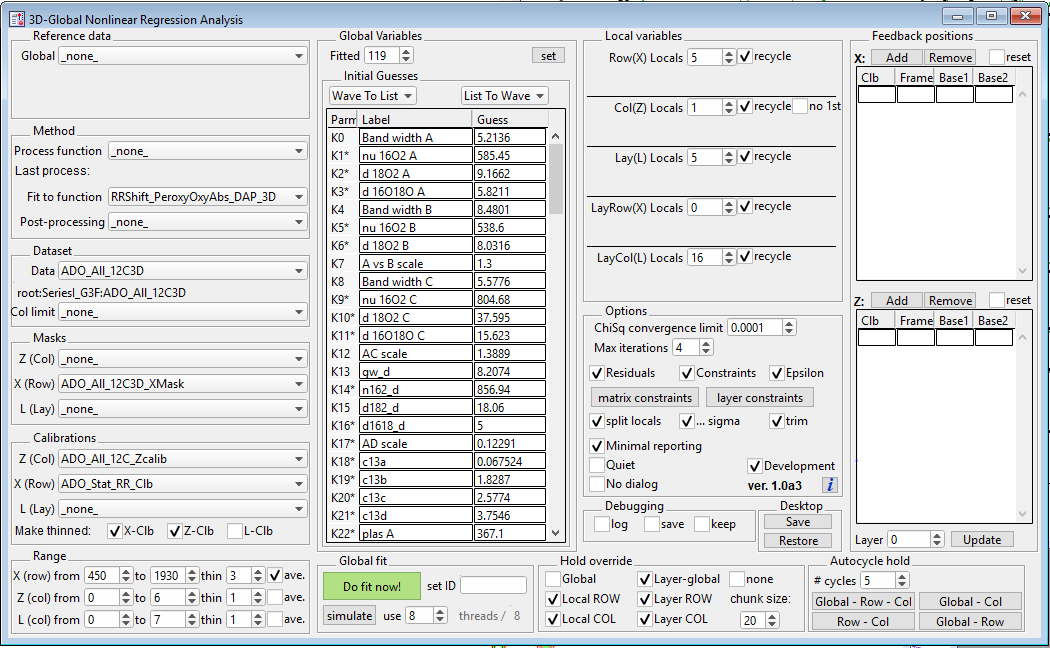

Supplement: Software repository [file NIHMS1559313-supplement-Software_repository.zip › G3F-master/Docs/G3FManualPictures/ControlPanel.png]

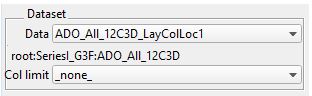

Supplement: Software repository [file NIHMS1559313-supplement-Software_repository.zip › G3F-master/Docs/G3FManualPictures/DataSet_Select.JPG]

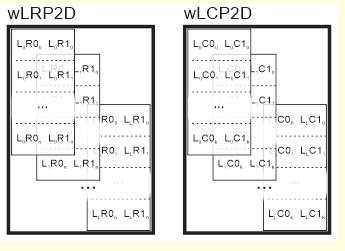

Supplement: Software repository [file NIHMS1559313-supplement-Software_repository.zip › G3F-master/Docs/G3FManualPictures/DataStructure_2.png]

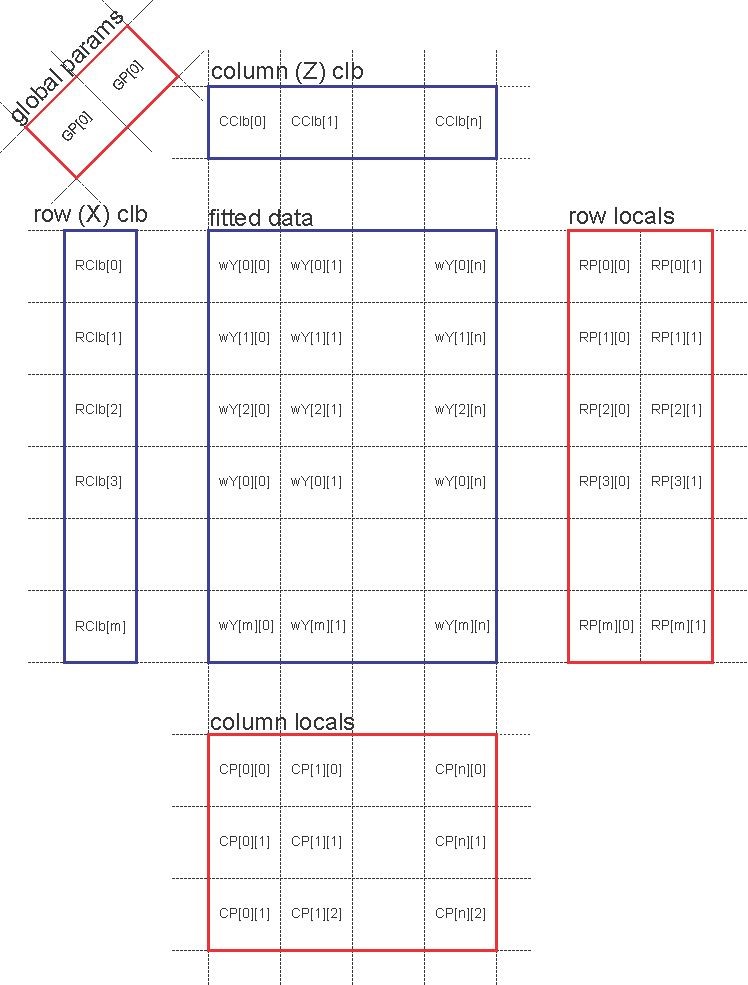

Supplement: Software repository [file NIHMS1559313-supplement-Software_repository.zip › G3F-master/Docs/G3FManualPictures/DataStructure_3.jpg]

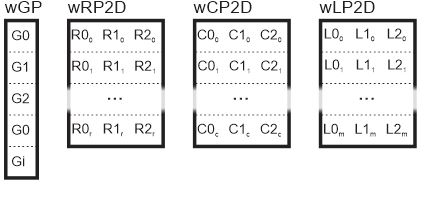

Supplement: Software repository [file NIHMS1559313-supplement-Software_repository.zip › G3F-master/Docs/G3FManualPictures/DataStructure_Simple.JPG]

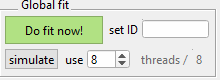

Supplement: Software repository [file NIHMS1559313-supplement-Software_repository.zip › G3F-master/Docs/G3FManualPictures/DoFit_Button.png]

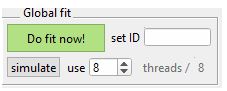

Supplement: Software repository [file NIHMS1559313-supplement-Software_repository.zip › G3F-master/Docs/G3FManualPictures/DoFit_Select.JPG]

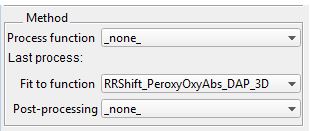

Supplement: Software repository [file NIHMS1559313-supplement-Software_repository.zip › G3F-master/Docs/G3FManualPictures/Function_Select.JPG]

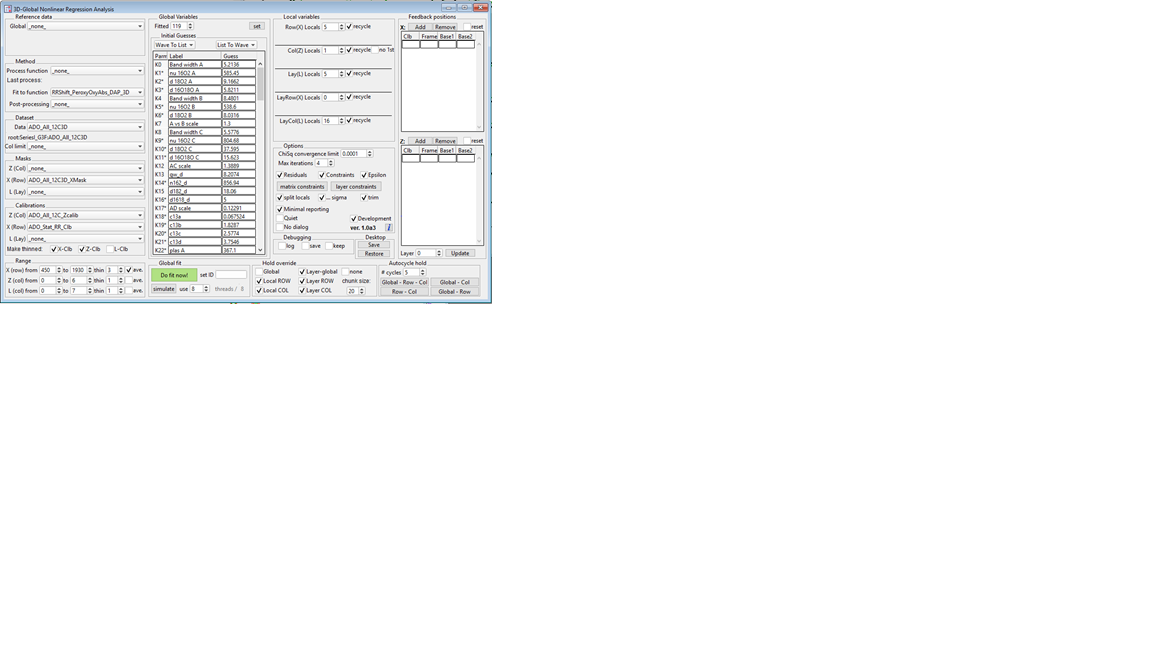

Supplement: Software repository [file NIHMS1559313-supplement-Software_repository.zip › G3F-master/Docs/G3FManualPictures/G3F_Control_Panel.png]

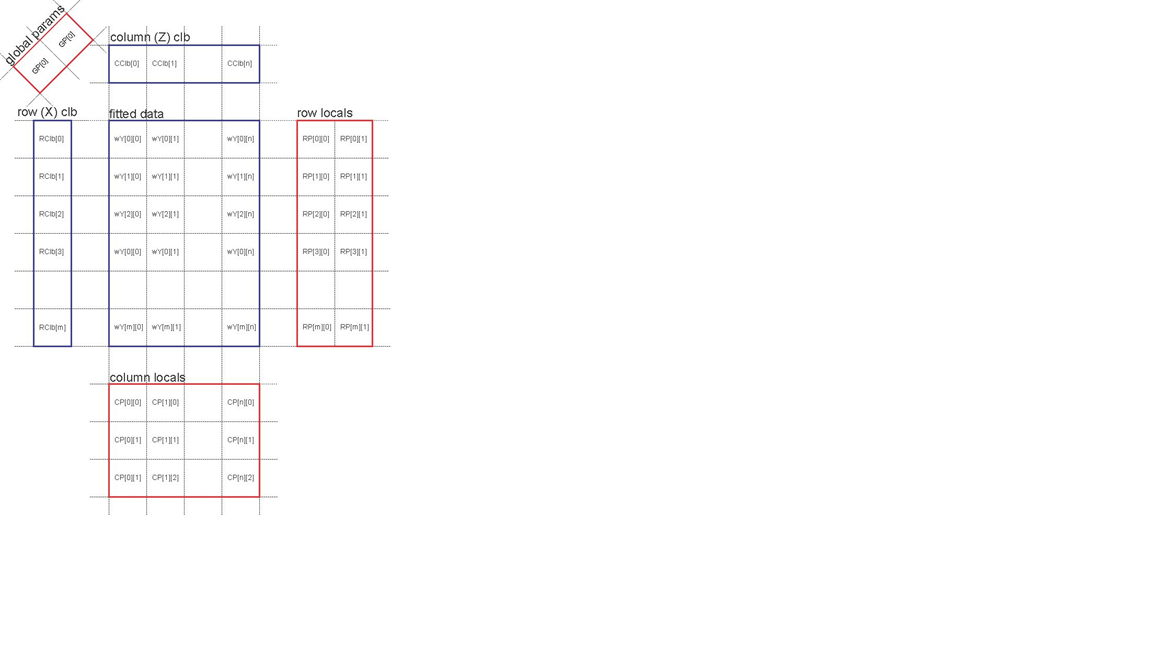

Supplement: Software repository [file NIHMS1559313-supplement-Software_repository.zip › G3F-master/Docs/G3FManualPictures/G3F_DataStructure_B.png]

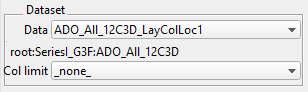

Supplement: Software repository [file NIHMS1559313-supplement-Software_repository.zip › G3F-master/Docs/G3FManualPictures/LoadData_1.png]

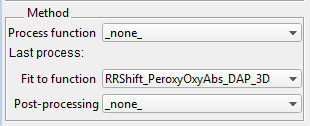

Supplement: Software repository [file NIHMS1559313-supplement-Software_repository.zip › G3F-master/Docs/G3FManualPictures/LoadFunction.png]

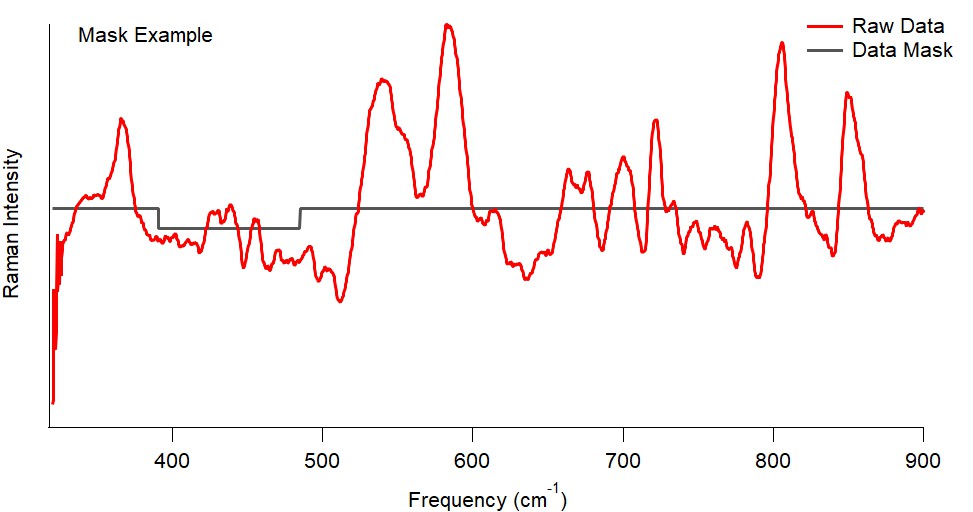

Supplement: Software repository [file NIHMS1559313-supplement-Software_repository.zip › G3F-master/Docs/G3FManualPictures/Mask_Example.jpg]
